# Clinical risk scores for the early prediction of severe outcomes in patients hospitalized for COVID-19

**DOI:** 10.1007/s11739-020-02617-4

**Published:** 2021-02-23

**Authors:** Walter Ageno, Chiara Cogliati, Martina Perego, Domenico Girelli, Ernesto Crisafulli, Francesca Pizzolo, Oliviero Olivieri, Marco Cattaneo, Alberto Benetti, Elena Corradini, Lorenza Bertù, Antonello Pietrangelo, List of contributors, Lucia Maria Caiano, Federica Magni, Elisabetta Tombolini, Chiara Aloise, Francesca Maria Casanova, Benedetta Peroni, Andrea Ricci, Stefania Scarlini, Ivan Silvestri, Matteo Morandi, Sara Pezzato, Francesca Stefani, Virginia Trevisan

**Affiliations:** 1 University of Insubria, Varese, Italy; 2 Ospedale Sacco, Milan, Italy; 3 Università Degli Studi Di Verona, Verona, Italy; 4 Ospedale San Paolo E Università Degli Studi Di Milano, Milan, Italy; 5 Università Di Modena E Reggio Emilia, Modena, Italy

**Keywords:** SARS-CoV 2, COVID-19, Risk prediction model, Respiratory failure

## Abstract

Coronavirus disease of 2019 (COVID-19) is associated with severe acute respiratory failure. Early identification of high-risk COVID-19 patients is crucial. We aimed to derive and validate a simple score for the prediction of severe outcomes. A retrospective cohort study of patients hospitalized for COVID-19 was carried out by the Italian Society of Internal Medicine. Epidemiological, clinical, laboratory, and treatment variables were collected at hospital admission at five hospitals. Three algorithm selection models were used to construct a predictive risk score: backward Selection, Least Absolute Shrinkage and Selection Operator (LASSO), and Random Forest. Severe outcome was defined as the composite of need for non-invasive ventilation, need for orotracheal intubation, or death. A total of 610 patients were included in the analysis, 313 had a severe outcome. The subset for the derivation analysis included 335 patients, the subset for the validation analysis 275 patients. The LASSO selection identified 6 variables (age, history of coronary heart disease, CRP, AST, D-dimer, and neutrophil/lymphocyte ratio) and resulted in the best performing score with an area under the curve of 0.79 in the derivation cohort and 0.80 in the validation cohort. Using a cut-off of 7 out of 13 points, sensitivity was 0.93, specificity 0.34, positive predictive value 0.59, and negative predictive value 0.82. The proposed score can identify patients at low risk for severe outcome who can be safely managed in a low-intensity setting after hospital admission for COVID-19.

## Introduction

In late 2019, the world started to face a new pandemic disease, the Coronavirus Disease of 2019 (COVID-19) caused by the Severe Acute Respiratory Syndrome CoronaVirus 2 (SARS-CoV 2). Interstitial pneumonia is the most important clinical manifestation of COVID-19, leading to severe acute respiratory failure and high mortality rates [1, 2].

The rapid and devastating onset of this disease required a re-organization of hospitals and healthcare facilities, with ordinary wards managing less severe COVID-19 patients and with a progressive increase in the number of Intensive Care Unit (ICU) beds. However, given the unexpectedly high rates of patients requiring non-invasive ventilation, also Internal Medicine wards were re-organized for the management of higher intensity patients.

Several studies reported typical patterns of laboratory tests in patients with COVID-19 and some independent predictors of disease severity and mortality, such as C-reactive protein (CRP), ferritin, and D-dimer, were identified [1, 3–5]. Likewise, individual patient characteristics such as advanced age and male sex, and comorbidities such as obesity, hypertension, diabetes, and coronary heart disease were found to be associated with mortality in COVID-19 patients [6–10].

The possibility to apply a clinical prediction model which includes factors associated with patient prognosis could allow clinicians to stratify COVID-19 patients and to rapidly identify the optimal management strategy. Two models including clinical, laboratory, and imaging variables were proposed by two Chinese groups of researchers, but none has been successfully implemented in clinical practice, as of yet [11, 12]. We aimed to derive and validate a new simple score using three different statistical approaches to be used for the early stratification of COVID-19 patients.

## Methods

A retrospective, observational, multicentre registry of patients hospitalized for COVID-19 in Italian hospitals was designed and promoted by the Italian Society of Internal Medicine (SIMI). Five centers participating in this registry contributed to the present study, two in Milan, one in Varese, one in Verona, one in Modena, Italy. As the registry aimed to record standard local practices, no specific treatments, tests, or procedures were mandated by the study protocol. All participating centers received approval from the local Ethics Committees.

Each participating center enrolled patients with a diagnosis of COVID-19 aged 18 years or older admitted to the Emergency Department or to a Medical Ward directly from the Emergency Department between February 17th and May 8th 2020. All patients were followed up for the duration of hospitalization. All data were collected using electronic medical records and gathered in an anonymized case report form (CRF). The completeness and accuracy of data collected from the patient medical records were checked by the registry-coordinating center.

Nasopharyngeal and oropharyngeal swab was collected on the day of admission or in the morning of the day after. Specimen analysis was carried out with reverse-transcriptase polymerase chain reaction (RT-PCR) method.

For the purpose of this study, information on demographic variables (age and sex), delay from symptoms onset to hospitalization, and medical history (hypertension, diabetes, chronic obstructive pulmonary disease, and coronary heart disease) was collected. Data on the following laboratory findings were included: white blood cells, lymphocytes, neutrophils, alanine transaminase (ALT), aspartate aminotransferase (AST), serum creatinine, D-dimer, and CRP levels were included. These data were acquired by physicians and were the results of an examination on the first day after admission.

Information on patient outcomes was collected until discharge. Severe outcome was defined as the composite of need for non-invasive ventilation, need for orotracheal intubation, or death, whichever came first. All other patients were classified as having a non-severe outcome.

### Statistical methods

Categorical variables are expressed as frequencies and percentage; continuous variables as mean and standard deviation or as medians and interquartile range, as appropriate.

Due to the high correlation between white blood cells, lymphocytes and neutrophils, we considered as potential predictors only white blood cells and neutrophils to lymphocytes ratio.

We used multiple imputation to deal with missing data. The missing values of all covariates were imputed by assuming that data were missing at random with 20 imputations; discriminant function and predictive mean matching were applied to impute binary responses and continuous variables, respectively.

To build the score, we considered as development cohort the patients belonging to the centers of Varese and Milan and as validation cohort the patients from Verona and Modena (geographical validation).

For the continuous predictors, the relationship with outcome was studied. We founded that linear relationship was a good approximation for white blood cells, ALT and creatinine; for the others, we utilized restricted cubic spline to assess optimal cut-offs.

Selection of predictors was made using three different techniques: (a) multivariate logistic regression with backward selection, (b) penalized logistic regression (Least Absolute Shrinkage and Selection Operator, LASSO, method), and (c) Random Forest (variable selection based on accuracy, mean minimal depth and times a root parameter). All strategies started with the full model.

Odds Ratio together with 95% confidence interval for derivation, validation and complete datasets were computed. The Akaike information criterion, Schwarz criterion and area under the curve (AUC) were evaluated for each model and the best model was chosen by comparing these criteria.

Score assignment for each predictor variable was found on its associated regression coefficient.

In addition, for each risk scores, receiver operating characteristics (ROC) curves were displayed and sensitivity, specificity, positive and negative predictive values calculated.

Model's calibration was assessed by Hosmer–Lemeshow C-test.

Analyses were conducted using SAS (Version 9.4, SAS Insitute, Cary, NC) and R (R Core Team, 2015).

## Results

A total of 610 patients were included in the analysis, 313 (51.3%) had a severe outcome and 297 (48.7%) had a non-severe outcome. Of patients with severe outcome, 145 required non-invasive ventilation (and 45 of them subsequently died), 39 required intubation (and 7 of them died), and a total of 181 patients died. The subset for the derivation analysis included 335 patients, of whom 173 (51.6%) had a severe outcome; the subset for the validation analysis included 275 patients, 140 with a severe outcome (50.9%). Baseline characteristics, comorbidities, laboratory results for each group are reported in Table 1. Briefly, in both derivation and validation cohorts patients with severe outcomes were older than patients with non-severe outcomes and the prevalence of male sex, comorbidities, as well as the levels of most laboratory values, with the exception of ALT and albumin, tended to be higher. The validation cohort was older (mean age 72 vs. 65 years), had more hypertensive patients (59% vs. 43%), and higher mean values of PCR (13.1 vs. 9.8 mg/L), creatinine (1.3 vs. 1.1 mg/dL) and D-dimer (1115 vs. 917) than the derivation cohort. However, the Hosmer–Lemeshow test showed a good calibration, which suggests that these differences did not impact the score.

**Table 1:** Baseline characteristics of the study participants—multiple imputation

		Derivation				Validation			
		Non-severe (*N* = 162)		Severe (*N* = 173)		Non-severe (*N* = 135)		Severe (*N* = 140)	
Age (years)	Mean (sd)	61.1	15.9	68.7	14.3	66.6	14.8	78.0	12.0
Onset time (days)	Median (iqr)	8	5–12	8	6–11	8	8–11	6	4–9
Sex—male	*N* (%)	92	56.8	105	60.7	81	60.9	94	67.6
Hypertension	*N* (%)	65	40.1	80	46.2	72	53.7	90	64.3
Diabetes	*N* (%)	25	15.4	31	17.9	17	12.6	46	32.9
COPD	*N* (%)	15	9.3	21	12.1	10	7.4	17	12.1
CHD	*N* (%)	34	21.0	58	33.5	26	19.3	46	32.9
WBC (10^9/L)	Mean (sd)	7.4	4.7	8.9	10.9	6.4	2.8	9.4	6.5
Neutrophils (10^9^/L)	Mean (sd)	5.6	4.8	6.7	4.3	4.8	3.0	7.4	5.3
Lymphocytes (10^9^/L)	Mean (sd)	1.3	1.2	1.7	8.0	1.2	0.7	1.0	1.1
N/L ratio	Mean (sd)	6.6	8.1	8.8	8.9	5.3	4.8	12.2	29.4
PCR (mg/L)	Mean (sd)	6.7	7.1	12.7	9.1	9.3	12.8	16.8	20.2
ALT (U/L)	Mean (sd)	41.0	29.0	43.9	40.6	40.6	43.6	41.4	72.2
AST (U/L)	Mean (sd)	45.8	31.8	64.7	46.3	48.2	35.2	57.1	64.2
Albumin (g/dL)	Mean (sd)	34.3	7.1	34.0	8.7	35.7	5.9	31.3	6.9
Creatinine (mg/dL)	Mean (sd)	1.1	0.8	1.2	0.8	1.0	1.0	1.6	1.5
D-dimer (mg/L)	Median (iqr)	716	439–1246	1325	679–2839	979	610–1832	1277	593–2770

OUTCOME 1: Severe = CPAP, IOT or Death

### Selection of predictors

Using Backward selection analysis, the following variables predicted severe outcome: CRP levels greater than 2.0 mg/dL, AST greater than 35 U/L, and D-dimer equal to or greater than 917 μg/L (Table 2). Overall, the area under the curve was 0.72; 0.78 in the derivation set and 0.66 in the validation set.

**Table 2:** Backward selection—multivariate logistic regression

	Derivation set		Validation set		All	
	OR	CI 95%	OR	CI 95%	OR	CI 95%
PCR (mg/dL)						
< 2.0	1.0	Ref	1.0	Ref	1.0	Ref
2–4.6	3.12	1.13–8.61	0.99	0.35–2.75	1.97	1.68–2.30
4.7–7.3	7.21	2.64–19.71	2.40	0.87–6.64	4.62	3.95–5.41
> = 7.4	8.87	3.42–22.99	3.22	1.34–7.74	5.92	5.15–6.81
AST (U/L)						
< 27	1.0	Ref	1.0	Ref	1.0	Ref
27–34	1.41	0.56–3.54	1.28	0.55–2.97	1.09	0.95–1.25
35–43	1.43	0.58–3.55	1.94	0.84–4.47	1.44	1.26–1.64
> = 43	3.36	1.54–7.33	1.94	0.97–3.90	2.47	2.21–2.77
D-dimer (mg/L)						
< 425	1.0	Ref	1.0	Ref	1.0	Ref
425–617	0.67	0.27–1.67	0.68	0.25–1.82	0.77	0.67–0.89
618–917	0.50	0.20–1.24	0.52	0.21–1.30	0.76	0.66–0.87
> = 917	1.54	0.71–3.36	0.74	0.35–1.54	1.24	1.11–1.39
Fit parameter						
AIC	394.86		376.27		14,904.54	
SC	433.0		412.43		14,978.63	
AUC	0.78		0.66		0.72	

Multiple imputation data*

*For variables with no missing data original data were used

AIC = Akaike Information Criterion

SC = Schwarz’s criterion

AUC = area under the curve

**Fig. 1:**
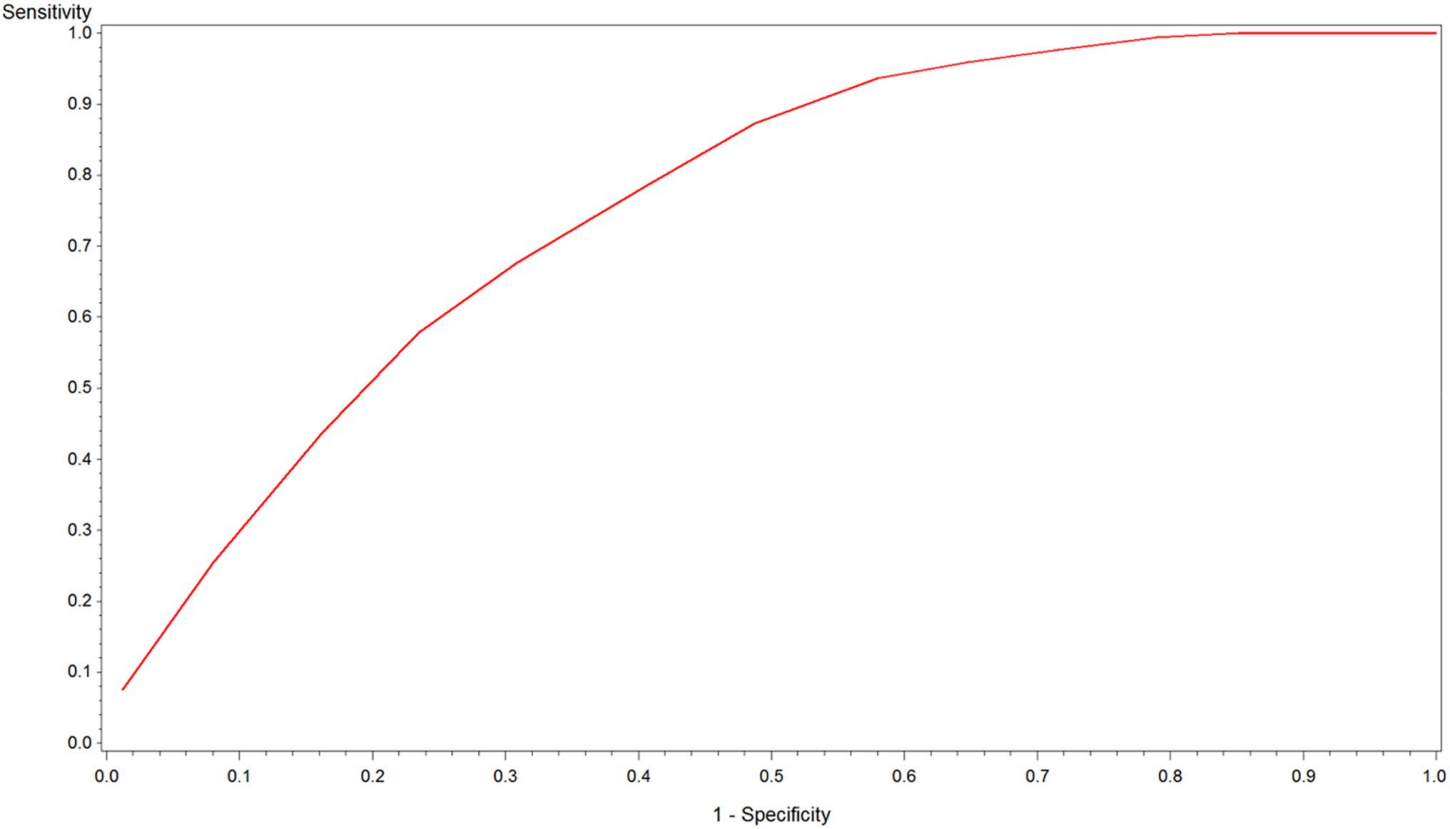
Area under the curve of the SIMI score (Lasso selection)

The LASSO selection identified 6 variables that predicted severe outcome: age older than 50 years, history of coronary heart disease, CRP levels greater than 2.0 mg/dL, AST greater than 35 U/L, D-dimer equal to or greater than 917 μg/L, and neutrophil/lymphocyte ratio greater than 3.3 (Table 3). Overall, the area under the curve was 0.76; 0.79 in the derivation set and 0.80 in the validation set (Fig. 1).

**Table 3:** LASSO selection—multivariate logistic regression

	Derivation set		Validation set		All	
	OR	CI 95%	OR	CI 95%	OR	CI 95%
Age (years)						
< 50	1.0	Ref	1.0	Ref	1.0	Ref
50–59	1.19	0.50–2.87	2.15	0.45–10.19	1.28	1.09–1.51
60–69	1.40	0.57–3.39	2.03	0.44–9.39	1.36	1.17–1.60
≥ 70	1.27	0.53–3.05	10.48	2.57–42.70	2.69	2.33–3.11
CHD	1.54	0.82–2.93	1.71	0.89–3.29	1.37	1.24–1.51
PCR (mg/dL)						
< 2.0	1.0	Ref	1.0	Ref	1.0	Ref
2–4.6	2.82	0.99–8.04	1.15	0.37–3.60	1.82	1.54–2.14
4.7–7.3	6.56	2.32–18.57	3.43	1.07–10.94	4.06	3.44–4.78
≥ 7.4	7.70	2.78–21.30	3.13	1.16–8.45	4.06	3.49–4,72
AST (U/L)						
< 27	1.0	Ref	1.0	Ref	1.0	Ref
27–34	1.44	0.56–3.72	1.17	0.45–3.02	1.11	0.96–1.28
35–43	1.45	0.57–3.71	2.22	0.85–5.79	1.38	1.20–1.59
≥ 43	3.43	1.53–7.72	2.08	0.94–4.59	2.44	2.17–2.75
D-Dimer (mg/L)						
< 425	1.0	Ref	1.0	Ref	1.0	Ref
425–617	0.59	0.23–1.52	0.41	0.13–1.26	0.70	0.60–0.82
618–917	0.49	0.19–1.25	0.29	0.10–0.85	0.65	0.56–0.75
≥ 917	1.28	0.56–2.94	0.31	0.13–0.76	0.88	0.78–0.99
N/L ratio						
< 2.3	1.0	Ref	1.0	Ref	1.0	Ref
2.3–3.3	1.03	0.39–2.70	0.51	0.17–1.54	0.92	0.79–1.08
3.3–5.0	2.05	0.78–5.40	0.90	0.35–2.34	1.50	1.30–1.73
≥ 5.0	1.30	0.55–3.07	1.64	0.69–3.89	1.78	1.56–2.02
Fit parameter						
AIC	402.61		335.91		14,208.38	
SC	467.45		397.39		14,334.34	
AUC	0.79		0.80		0.76	

Multiple imputation data*

*For variables with no missing data original data were used

AIC = Akaike Information Criterion

SC = Schwarz’s criterion

AUC = Area under the curve

Finally, the Random Forest selection identified the following three variables: CRP greater than 2.0 mg/dL, AST greater than 35 U/L, and N/L ratio greater than 3.3 (Table 4). Overall, the area under the curve was 0.72; 0.75 in the derivation cohort and 0.70 in the validation cohort.

**Table 4:** Random Forest selection—multivariate logistic regression

	Derivation set		Validation set		All	
	OR	CI 95%	OR	CI 95%	OR	CI 95%
PCR (mg/dL)						
< 2.0	1.0	Ref	1.0	Ref	1.0	Ref
2–4.6	3.02	1.09–8.38	0.92	0.33–2.62	1.81	1.55–2.13
4.7–7.3	6.40	2.32–17.64	1.93	0.69–5.41	3.73	3.19–4.37
≥ 7.4	8.51	3.24–22.37	2.39	0.97–5.89	4.27	3.70–4.93
AST (U/L)						
< 27	1.0	Ref	1.0	Ref	1.0	Ref
27–34	1.32	0.53–3.26	1.01	0.43–2.39	1.03	0.90–1.19
35–43	1.59	0.64–3.92	1.64	0.70–3.88	1.44	1.26–1.64
≥ 43	3.04	1.41–6.54	1.58	0.77–3.23	2.38	2.13–2.67
N/L ratio						
< 2.3	1.0	Ref	1.0	Ref	1.0	Ref
2.3–3.3	0.95	0.38–2.42	0.72	0.27–1.93	0.98	0.85–1.14
3.3–5.0	2.26	0.90–5.89	1.06	0.45–2.46	1.58	1.38–1.82
≥ 5.0	1.52	0.67–3.44	2.39	1.13–5.07	2.25	1.99–2.55
Fit parameter						
AIC	402.70		365.45		14,750.53	
SC	440.84		401.62		14,824.62	
AUC	0.75		0.70		0.72	

Multiple imputation data*

*For variables with no missing data original data were used

AIC = Akaike Information Criterion

SC = Schwarz’s criterion

AUC = Area under the curve

### Selection of the predictive scores

Based on the results of the multivariate analyses, a score was built after each method was used. After backward selection analysis, we obtained a score that included three variables and a total of 11 points (Table 6 available in the Appendix). Using a cut-off of six in the validation set, the score resulted in a sensitivity of 0.88, a specificity of 0.34, a positive predictive value of 0.58 and a negative predictive value of 0.73.

After the LASSO selection, we obtained a score with six variables and a total of 13 points (Table 5 and Table 6 in the Appendix). Using a cut-off of seven in the validation set, the score resulted in a sensitivity of 0.93, a specificity of 0.34, a positive predictive value of 0.59 and a negative predictive value of 0.82.

**Table 5:** SIMI score

	Value	Score
Age (years)	< 50	0
	50–69	1
	≥ 70	3
CHD		1
PCR (mg/dL)	< 2.0	0
	2–4.6	2
	≥ 4.7	4
AST (U/L)	< 27	0
	27–43	1
	≥ 43	2
D-dimer (mg/L)	< 917	0
	≥ 917	1
N/L ratio	< 3.3	0
	3.3–5.0	1
	≥ 5.0	2

A score < 7 is associated with a non-severe outcome, a score of 7 or more is associated with a severe outcome

With Random Forest selection, a score with three variables and a total of nine points was obtained (Table 6 in the Appendix). Using a cut-off of five in the validation set the score resulted in a sensitivity of 0.86, a specificity of 0.38, a positive predictive value of 0.59 and a negative predictive value of 0.73.

## Discussion

Using the database of an Italian registry, we aimed to derive and validate a simple score to stratify the risk of adverse outcomes in patients hospitalized for COVID-19. After comparing three different statistical approaches, the score obtained using the LASSO selection resulted as the best performing score, with an area under the curve of 0.80 in the validation cohort, a sensitivity of 0.93 and a negative predictive value of 0.82. The accuracy of the score in the validation set was nearly identical to that observed in the derivation set. With six variables, four of which obtained after laboratory testing, this score named SIMI score after the name of the society that promoted the registry (Società Italiana di Medicina Interna), may accurately identify lower risk patients who can be conservatively managed in lower intensity settings. In our cohort, these patients survived after hospitalization without requiring Continuous Positive Airway Pressure or orotracheal intubation.

COVID-19 has become an important challenge for health organizations in particular because of the need for intensive or sub-intensive care management for a relevant number of patients. Due to the lack of a sufficient number of beds in the Intensive Care Units, several Internal Medicine wards dedicated to COVID-19 patients were partially re-organized to manage patients requiring ventilatory support with non-invasive ventilatory support. During the peaks of the pandemic, it becomes crucial to preserve these beds for higher risk patients and to find alternative paths for lower risk patients, including early discharge or admission to dedicated post-acute facilities. In our study, these lower risk patients represented slightly less than 50% of the population. For the remaining, non-low-risk patients, a correct stratification may allow to more timely start adequate management strategies such as ventilatory support and pharmacologic treatment with the aim to reduce the need for Intensive Care Unit beds.

Two studies carried out in China have proposed risk assessment models for COVID-19 patients. A study from Wuhan included 377 patients and found age, neutrophils to lymphocytes ratio, CRP, and D-dimer as predictors of the severity of COVID-19, defined as severe pneumonia and non-severe pneumonia [11]. Severe pneumonia was defined by the presence of respiratory rate of greater than 30 breaths/min, severe respiratory distress, or SpO2 of less than 90% on room air. Patients with acute respiratory distress syndrome (ARDS), sepsis, or septic shock were also included in the definition. The proposed model resulted in a negative predictive value of 0.93, a positive predictive value of 0.41, a specificity of 0.70 and a sensitivity of 0.89. In a larger study from China including 1590 patients for the derivation set and 710 patients for the validation set, 10 variables were identified as independent predictive factors for adverse outcome defined as admission to the Intensive Care Unit, need for invasive ventilation, or death [12]. These variables included chest radiographic abnormalities, age, hemoptysis, dyspnea, unconsciousness, number of comorbidities, cancer history, neutrophils to lymphocytes ratio, lactate dehydrogenase, and direct bilirubin. The Area Under the Curve in the validation cohort was 0.88. The SIMI score has the advantage of a clear definition of the outcome, as in the latter score from China, and of the use of fewer variables, which potentially makes it more suited for daily clinical practice in the Emergency Room. A clinical prediction model with 8 variables was recently proposed by an Italian group [13]. Despite promising results, the model was tested on a small sample of patients and requires further validation.

This study has some limitations that need to be acknowledged. First, the sample size for the derivation set and the validation set was small and the results need to be confirmed in larger cohorts. Second, the study was conducted in a single country, and subsequent validation in different geographic areas with different health organizations would be required. Third, given the observational nature of the study, we may have missed other variables that are not routinely tested in these patients on admission and that may also result to be predictive of adverse outcomes. Finally, the clinical impact of this score also needs to be assessed in management studies which randomize patients or centers to the use of the score or to gestalt.

In conclusion, we here propose a simple score based on six variables to assist clinicians in the stratification of the risk of patients admitted for COVID-19. The SIMI score has a good accuracy, and, in particular, a good sensitivity to identify patients at low risk for adverse outcome during hospitalization. Future studies should assess the clinical impact of this score.

## Appendix

See Table 6.

**Table 6:** Scores derived from the three analyses

	Value	Score 1	Score 2	Score 3
Age (years)	< 50		0	
	50–59		1	
	60–69		1	
	≥ 70		3	
CHD			1	
PCR (mg/dL)	< 2.0	0	0	0
	2–4.6	2	1	2
	4.7–7.3	5	4	4
	≥ 7.4	5	4	4
AST (U/L)	< 27	0	0	0
	27–34	1	1	1
	35–43	1	1	1
	≥ 43	3	2	2
D-dimer (mg/L)	< 425	0	0	
	425–617	1	0	
	618–917	1	0	
	≥ 917	2	1	
N/L ratio	< 2.3		0	0
	2.3–3.3		0	0
	3.3–5.0		1	1
	≥ 5.0		2	2
